# Homogenous Fluorescent Assays for Characterizing Small-Molecule Activators of AMP-Activated Protein Kinase (AMPK)

**DOI:** 10.2174/1875397300801010034

**Published:** 2008-02-25

**Authors:** Laurie J Reichling, Steven M Riddle, Baigen Mei, Rica Bruinsma, Tony A Goossens, Kristin G Huwiler, Mark Maffitt, Alyssa M.G Newport, Xiao-Dong Qian, Carmen Ruttimann-Johnson, Kurt W Vogel

**Affiliations:** Invitrogen Discovery Sciences, 501 Charmany Dr, Madison, WI 53719

## Abstract

AMP activated protein kinase (AMPK) is a key regulator of cellular metabolism. AMPK activity is modulated in part by binding of AMP to the γ-subunit of the kinase, which increases the activity of the catalytic α-subunit. Because increased AMPK activity in the liver and in skeletal muscle leads to increased fatty acid oxidation and decreased cholesterol and fatty acid biosynthesis, activators of AMPK are being sought for treatment of type-2 diabetes and other metabolic disorders. The unique mechanism of AMPK activation offers an opportunity to develop small molecules that directly upregulate AMPK activity, and there exists a need for simplified methods to identify and characterize small-molecules that show isoform-specific effects on AMPK. We have developed a suite of fluorescence-based assays to identify and characterize such compounds, and have used these to characterize and compare activity of recombinant AMPK α_1_β_1_γ_1_ and α_2_β_1_γ_1_ isoforms in response to small molecule activators and inhibitors.

## INTRODUCTION

AMP-activated protein kinase (AMPK) plays a central role in regulating metabolic activity by downregulating ATP-consuming processes and upregulating catabolism in response to environmental stresses that decrease the cellular [ATP]:[AMP] ratio. Upon activation, AMPK phosphorylates and inactivates acetyl CoA carboxylase (ACC, involved in fatty acid biosynthesis), HMG-CoA reductase (a key regulator of cholesterol synthesis and the target of the statin class of drugs) [1], as well as glycogen synthase [2]. AMPK is a heterotrimeric complex consisting of α, β, and γ subunits [3, 4], and is directly regulated by at least three processes: activation through phosphorylation of Thr-172 in the activation loop of the catalytic α subunit by LKB1 [5-9] or CaMKKα or β [10, 11], deactivation *via *dephosphorylation at this site by PP2 [12], and allosteric activation *via *AMP [13].

AMPK is broadly expressed in a variety of tissues, with different isoforms being dominantly expressed in different tissues. The trimeric complex consists of α, β, and γ subunits, with two isoforms identified for each of the α and β subunits, and three identified for the γ subunit, giving rise to a possibility of twelve isoforms of AMPK. In liver, the α_1_ and α_2_ subunits are present in approximately equal amounts, with the β_1_ and γ_1_ being the predominant forms of their respective subunits [14]. In contrast, in heart and in skeletal muscle the α_2_ form predominates over the α_1_ form by a greater than a 2:1 ratio, and the β_2_ subunit is predominant over the β_1_ form in these tissues as well [15]. 

Therapeutic targeting of AMPK activation has been shown to have positive results in models of diabetes. The small molecule drug metformin has been used for approximately 50 years in the treatment of type 2 diabetes, and functions by indirectly promoting AMPK phosphorylation and activation [16]. Another small molecule, AICAR (5-aminoimidazole-4-carbozamide riboside), has proven to be a useful tool for pharmacologically activating AMPK without perturbing the cellular [ATP]:[AMP] ratio. AICAR is taken up into cells *via *the adenosine transport system and then phosphorylated to form AICAR monophosphate, or ZMP [17], which activates AMPK by binding to the allosteric AMP-binding site. Recently, the results of a highly miniaturized radiometric assay were reported, in which of a library of over 700,000 compounds was screened for AMPK activators or inhibitors [18], and optimization of hits led to a thienopyridone-based small molecule (A-769662) that activated rat-liver or recombinant AMPK (α_1_β_1_γ_1_) with an EC_50_ of approximately 800 nM, and showed positive effects in the treatment of diabetic *ob/ob* mice [19]. Interestingly, this compound was able to increase AMPK activity *in vitro* in the presence of saturating amounts of AMP, suggesting the possibility of multiple allosteric sites that can be exploited to develop compounds that activate AMPK. Additionally, AMPK activation has emerged as a therapeutic target for atherosclerosis and cancer [20].

In addition to therapeutic interest in AMPK activation, there is evidence that either direct inhibition [21] or a leptin-induced decrease [22, 23] in AMPK activity in the hypothalamus can reduce food intake and body weight. Compound C, a pyrazo[1,5-*a*]pyrimidine compound that inhibits AMPK was discovered in a high-throughput screen [16] and subsequently shown to decrease food intake in mice [21]. Expression of a dominant negative form of AMPK in mouse hypothalamus was shown to reduce both food intake and weight gain, with opposite results seen when a constitutively active form of AMPK was expressed [22].

**Fig. (1):**
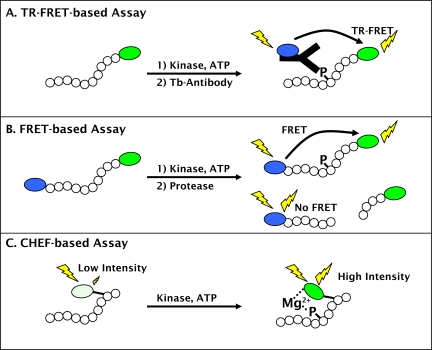
Schematic of fluorescent assay formats used to characterize AMPK activators of inhibitors. (**A**) The TR-FRET format detects association between fluorescein labeled, phosphorylated peptide and a terbium-labeled phosphospecific antibody. (**B**) The FRET-based format uses a peptide substrate terminally labeled with a coumarin-fluorescein FRET pair and measures the amount of phosphorylated product due to a decrease in sensitivity of the phosphorylated peptide to proteolysis. (**C**) The CHEF-based format uses a peptide substrate that incorporates the non-natural, fluorogenic Sox residue. Upon phosphorylation of a proximal serine, threonine, or tyrosine residue, the Sox moiety forms a Mg^2+^ mediated bridge between the Sox residue and the phosphate group and becomes fluorescent.

Because of the key role that AMPK plays in maintaining metabolic homeostasis and the diversity of isoforms that exist in different tissues, there is a need for simple, homogenous, non-radiometric methods to identify and characterize small-molecule modulators of AMPK activity. To address this need, we have developed a suite of assays (Fig. **1**) for different stages of the discovery process. We have developed a time resolved Förster resonance energy transfer (TR-FRET) assay that is well suited to HTS applications, due to its inherent resistance to common forms of assay interference such as colored, fluorescent, or precipitated compounds [24]. Further, we have developed secondary assays that provide a response that is directly proportional to the amount of product formed, and are therefore well suited to detailed mechanistic studies (or hit-confirmation) of small-molecule modulators of AMPK activity. The first of these formats is a FRET-based format in which a peptide substrate for AMPK is labeled on its termini with a coumarin / fluorescein FRET pair. After the kinase reaction, a site-specific protease is added that preferentially cleaves non-phosphorylated substrate, thereby decreasing the FRET signal that remains intact in the non-cleaved, phosphorylated product [25]. In this manner, changes in FRET can be directly correlated to substrate phosphorylation. The second format is based upon the principal of chelate-enhanced fluorescence (CHEF), in which phosphorylation of a serine, threonine, or tyrosine residue causes phosphate-dependent chelation of magnesium between the phosphate group and a non-natural, fluorogenic amino acid (Sox), that has been incorporated into the peptide sequence and that becomes fluorescent upon this chelating event [26]. The FRET-based assay format provides a convenient method for automated compound profiling, and the CHEF-based assay provides the ability to directly monitor purified AMPK activity in real-time.

## MATERIALS AND METHODS

### General Reagents

AMP, ZMP (5-Aminoimidazole-4-carboxamide-1-β-D-ribofuranosyl 5′-monophosphate), APR (adenosine 5′- monophosphoramidate) and ATMP (adenosine 5′-O-thiomonophosphate) were from Sigma-Aldrich (St Louis, MO). Compound C (6-[4-(2-Piperidin-1-yl-ethoxy)-phenyl)]-3-pyridin-4-yl-pyrrazolo [1,5-a] - pyrimidine) was from EMD Chemicals Inc (San Diego, CA). TR-FRET based assay reagents (commercialized under the LanthaScreen™ TR-FRET trade name) and FRET-based assay reagents (commercialized under the Z’-LYTE™ trade name) and CAMKK1 were from Invitrogen Discovery Sciences (Madison, WI). CHEF-based assay reagents (commercialized under the Omnia™ trade name) were from Biosource (Hopkinton, MA). SAMS peptide substrate was from Upstate.

### Cloning, Expression, and Purification of AMPK α_1_β_1_γ_1_ and α_2_β_1_γ_1._

Genes for the α_1_ (accession # NP_006242.4)_, _α_2_ (NP_006243.2)_, _and β_1_ (NP_006244.2) subunits were fused to the 3’ end of the GST gene found in pDEST20 *via *standard GATEWAY subcloning methods. The γ_1_ gene (NP_002724.1) was subcloned in pDEST10 in order to create a vector that would express a protein with an N-terminal His tag. Bacmid DNA was derived from all four vectors, and used to transfect insect cells. AMPK α_1_β_1_γ_1_ and α_2_β_1_γ_1_ were expressed in Sf9 insect cells using an MOI of 1 for each subunit. The cells were harvested 72 hours post infection and the cell paste was stored at -80°C until needed.

Both AMPK isoforms were purified similarly, and all steps were performed on ice or at 4°C unless otherwise stated. Cell paste was suspended in lysis buffer (20 mM Tris pH 8.0, 1% Triton X-100, 1 mM Na_3_VO_4_, 25 mM β-glycerophosphate, 1 mM PMSF, 5 mM benzamidine, 10 µg/mL leupeptin, 10 µM E-64, 2 mM EDTA, and 2 mM DTT), mixed with a hand-held homogenizer, and then clarified by centrifugation. The clarified lysate was then purified on glutathione sepharose 4B, and fractions containing significant amounts of AMPK as determined by SDS-PAGE were pooled and passed over a Superdex 200 column. Fractions were collected and pooled based on purity as assessed by SDS-PAGE and by activity as analyzed by a radiometric activity assay using SAMS peptide. AMPK was then concentrated to 0.3 mg/mL, and activated by incubation at room temperature for 2 hours with his-tagged CAMKK1 (0.1 mg per mg of AMPK), 1 mM ATP, 10 mM MgCl_2_, 10 µg/mL calmodulin, and 0.5 mM CaCl_2_, followed by further purification on glutathione sepharose 4B to remove CAMKK1. The purified enzyme was dialyzed into storage buffer (50 mM Tris pH 7.5, 50% glycerol, 150 mM NaCl, 0.02% Triton X-100, 0.5 mM EDTA, and 2 mM DTT) and stored at -80°C until use.

### Radiometric Assays

Kinase reactions were performed at room temperature in 50 mM HEPES pH 7.5, 0.01% BRIJ-35, 10 mM MgCl_2_, and 1 mM EGTA in a 30 µL assay volume in 96-well plates. For the inhibition experiments, a 2-fold dilution series of inhibitor stock solutions was first prepared in 100% DMSO at 100-fold concentration of inhibitor to be used in the assay. Prior to the assay, 4 µL of each inhibitor concentration was further diluted to 100 µL in assay buffer to prepare a working stock of inhibitor in 4% DMSO at 4-fold the final assay concentration. For the activation experiments, activators were prepared in 100% H_2_O at 4-fold the concentration to be tested in the assay. Assays were performed as follows: 7.5 µL of the inhibitor or activator working stock was added to a 17.5 µL solution of assay buffer, substrate and [^32^P]-ATP (0.5 µCi/well), followed by 5 µL of enzyme to start the assay. All assays were performed in triplicate. The final concentration of SAMS peptide substrate in the reaction was 200 µM. AMPK α_1_β_1_γ_1_ was used at 334 ng/mL (1.8 nM) in the inhibition experiments and 83.3 ng/mL (0.44 nM) in the activation experiments, at an ATP concentration of 35 µM. AMPK α_2_β_1_γ_1_ was used at 1670 ng/mL (8.9 nM) in the inhibition experiments and 267 ng/mL (1.4 nM) in the activation experiments, at an ATP concentration of 65 µM. After 10 minutes, 20 µL of the reaction was spotted onto a p81 filter. The filters were washed three times in 0.5% phosphoric acid, and twice in distilled water before being counted in Beckman Coulter Ready Safe liquid scintillation cocktail using a Beckman Coulter LS6500 Multipurpose Scintillation Counter. Scintillation Counter output (cpm) was used to calculate specific activity (nmole / (min•mg)).

### Time-Resolved FRET (TR-FRET) Activity Assays

Kinase reactions were performed in a 10 µL assay volume at room temperature in 384-well low volume plates (Corning model 3676) using the same assay buffer as the radiometric assay. Activators and inhibitors were prepared as described for the radiometric assay. Assays were performed as follows: 2.5 µL of the inhibitor or activator working stock was added to a 5 µL solution of kinase and substrate, followed by 2.5 µL of ATP to start the assay. Assays were performed in quadruplicate. The final concentration of fluorescein-labeled CREBtide peptide substrate (derived from residues 123-136 of CREB protein) in the reaction was 400 nM. AMPK α_1_β_1_γ_1_ was used at 600 ng/mL (3.1 nM) in the inhibition experiments and 200 ng/mL (1.1 nM) in the activation experiments, at an ATP concentration of 50 µM. AMPK α_2_β_1_γ_1_ was used at 5 µg/mL (26.5 nM) in the inhibition experiments and 600 ng/mL (3.2 nM) in the activation experiments, at an ATP concentration of 150 µM. After one hour, a 10 µL solution of EDTA and terbium labeled phospho-specific antibody in 20 mM Tris, pH 7.5 and 0.01% NP-40 was added to a final well volume of 20 µL, a final antibody concentration of 1 nM, and a final EDTA concentration of 10 mM. After a 30 minute incubation at room temperature the assay plate was read on a BMG Pherastar plate reader using the LanthaScreen™ filter module. The TR-FRET ratio was calculated as the intensity of the acceptor signal divided by the intensity of the donor signal.

### FRET-based Kinase Activity Assays

Kinase reactions were performed in a 10 µL assay volume at room temperature in 384-well low volume plates (Corning model 3676) using the same assay buffer as the radiometric assay. Inhibitors and activators were prepared as described for the radiometric assays. Assays were performed as follows: 2.5 µL of the inhibitor or activator working stock was added to a 5 µL solution of kinase and substrate, followed by 2.5 µL of ATP to start the assay. All assays were performed in quadruplicate. The final concentration of Z’-LYTE™ S/T23 peptide substrate in the reaction was 2 µM. AMPK α_1_β_1_γ_1_ was used at 79 ng/mL (0.42 nM) in the inhibition experiments and 41 ng/mL (0.22 nM) in the activation experiments, at an ATP concentration of 50 µM. AMPK α_2_β_1_γ_1_ was used at 177 ng/mL (0.92 nM) in the inhibition experiments and 41 ng/mL (0.22 nM) in the activation experiments, at an ATP concentration of 150 µM. After one hour, 5 µL of protease solution was added to a final well volume of 15 µL. After a 1 hour incubation at room temperature the assay plate was read on a Tecan Safire plate reader using an excitation wavelength of 400 nm (12 nm bandpass), and emission wavelengths of 445 nm (12 nm bandpass) and 520 nm (12 nm bandpass). The FRET ratio was calculated as the intensity of the donor signal divided by the intensity of the acceptor signal. The extent of substrate phosphorylation was calculated from the FRET ratio as described previously [25].

### Chelation Enhanced Fluorescence (CHEF)-Based Activity Assays

Kinase reactions were performed in a 50 µL reaction volume at 30°C in 96-well half-area plate (Corning Model 3992) using the same assay buffer as the radiometric assays. Inhibitors and activators were prepared as described for the radiometric assays. Assays were performed by mixing 10 µL enzyme solution, 10 µL of substrate peptide solution, 10 µL of AMP solution (or H_2_O for the activator experiments), and 10 µL of inhibitor or activator solution. The reactions were initiated by adding 10 µL of ATP solution. The final concentration of Omnia™ S/T23 peptide substrate in the reaction was 8 µM. AMPK α_1_β_1_γ_1_ was used at 320 ng/mL (1.7 nM), at an ATP concentration of 40 µM. AMPK α_2_β_1_γ_1_ was used at 640 ng/mL (3.4 nM), at an ATP concentration of 120 µM. The concentration of AMP in the inhibitor titrations was 80 µM. Immediately after addition of ATP the assay plate was placed into a fluorescence plate reader (Molecular Devices SpectraMax M5, pre-warmed to 30°C). The plates were read for 60 minutes using an excitation wavelength of 360 nm and emission wavelength of 485 nm. The reaction rates were calculated as the change in fluorescence intensity over time (Rfu/sec) over the period where the assay response was linear.

### Data Analysis

Data analysis and curve-fitting was performed using GraphPad Prism software (GraphPad Software, Inc, San Diego CA). Curves were fit using a 4-parameter non-linear regression, with the “top” of the curve set to a fixed value such that datapoints prior to a decrease in AMPK activity (due to competition at the ATP site) showed minimal deviation from the calculated curve.

## RESULTS AND DISCUSSION

### Expression and Purification of Recombinant Human AMPK α_1_β_1_γ_1_ and α_2_β_1_γ_1_ from Insect Cells

Structural studies of the heterotrimer have demonstrated that the α and β subunits interact with one another *via *their C-termini [27]. The C-terminus of the β subunit also interacts with the γ subunit, but the γ subunit contact points are more centrally located [28, 29]. The former observation suggested that attaching large purification tags (e.g. GST) to the α and β C-termini could be contraindicated. The latter observation was taken as an indication that a small tag (e.g. 6x His) would be prudent irrespective of the tag location. Indeed, various permutations of tagged subunits were tested, and those discussed here were the only combinations that gave enzymatically active heterotrimeric protein (data not shown).

**Fig. (2):**
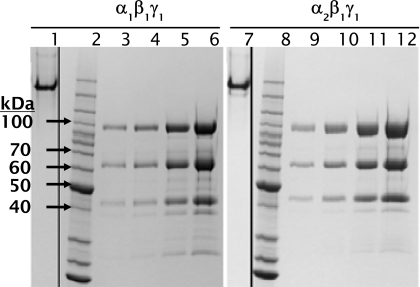
SDS-PAGE and native gel analysis of AMPK α_1_β_1_γ_1_ and α_2_β_1_γ_1_. Lanes 1 and 7: native (non-denaturing) PAGE samples. Lanes 2 and 8: Invitrogen Benchmark protein ladder. Lanes 3 – 6: AMPK α_1_β_1_γ_1_ loaded at 1, 2, 5, or 10 µg / lane. Lanes 9 – 12: AMPK α_2_β_1_γ_1_ loaded at 1, 2, 5, or 10 µg / lane. The GST-tagged α_1_ and α_2_ subunits have calculated molecular weights of 90.8 and 91.4 kDa, respectively. The GST-tagged β_1_ and 6-His-tagged γ_1_ subunits have calculated molecular weights of 57.8 and 41.7 kDa, respectively.

**Fig. (3):**
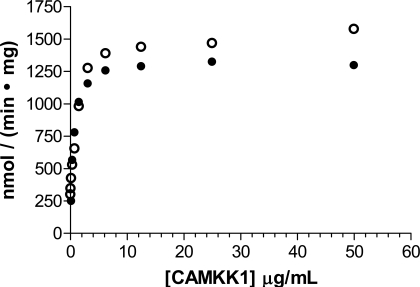
Activation of AMPK α_1_β_1_γ_1_ (●) and AMPK α_2_β_1_γ_1_ (○) by CAMKK1. Activity of AMPK was determined after a 2 hour incubation with CAMKK1 at room temperature.

Both AMPK isoforms could be purified to approximately 80% purity as assessed by SDS-PAGE by a single purification step on GSH-sepharose, and further purification by sizing column yielded material that was > 85% pure by SDS-PAGE analysis. Purified recombinant AMPK ran as a single distinct band by native PAGE analysis, indicating that all three AMPK subunits expressed in insect cells form a stable heterotrimeric complex (Fig. **2**). In contrast to trimeric AMPK expressed in *E coli*, which showed no detectable activity until activated by an upstream kinase [30], trimeric AMPK expressed and purified from insect cells showed measurable activity that could be increased 5- to 6- fold with CAMKK1 (Fig. **3**). Both α_1_β_1_γ_1_ and α_2_β_1_γ_1_ isoforms were activated to a similar level of activity using either CAMKK1 or LKB1/STRADα/MO25α (data not shown).

### ATP K_m_ Determination

**Fig. (4):**
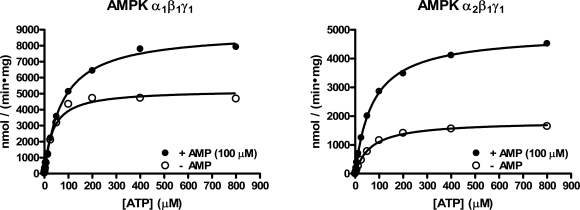
Radiometric assay of AMPK α_1_β_1_γ_1_ and α_2_β_1_γ_1_ in the presence or absence of AMP.

ATP K_m_ determinations were performed for both AMPK isoforms in the radiometric assay format using saturating concentrations of SAMS substrate in the presence or absence of 100 µM AMP (Fig. **4**). The α_1_β_1_γ_1_ isoform showed an ATP K_m_ of 34 µM in the absence of AMP, and 74 µM in the presence of AMP (Table **1**). The α_2_β_1_γ_1_ isoform showed similar values of 65 µM and 73 µM in the absence or presence of AMP, respectively. These values are similar to those previously reported for purified rat liver AMPK, which were 86 µM or 70 µM in the absence or presence of AMP, respectively [31], and were on the order of the apparent ATP K_m_ values determined in the fluorescent assay formats (Table **1**).

### Comparison of Small-Molecule Modulators on AMPK α_1_β_1_γ_1_ and α_2_β_1_γ_1_ Activity

Compound C was originally described as the first “specific” inhibitor of AMPK [16], but has recently been shown to inhibit a range of other kinases [32, 33]. In our hands, compound C showed a slight (< 3-fold) specificity towards the α_2_β_1_γ_1_ isoform in all assay formats tested (Table **1**), with IC_50_ values on the order of the previously reported 109 nM against rat liver AMPK [16], as well as the slightly higher value shown in other studies [34].

**Fig. (5):**
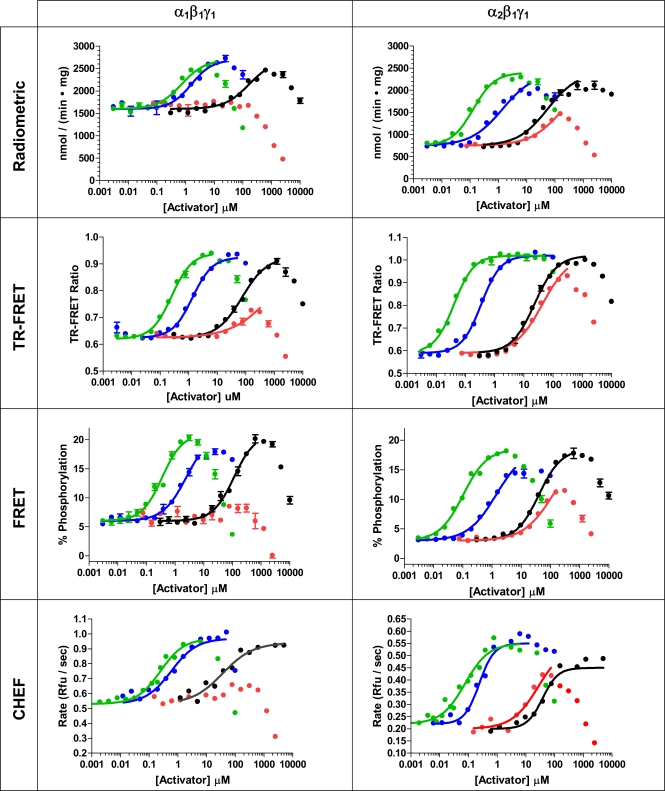
Small-molecule activation of AMPK α_1_β_1_γ_1_ and α_2_β_1_γ_1_ as determined in different assay formats. AMP (blue circles), ZMP (black circles), ATMP (green circles), APR (red circles).

AMPK activation experiments were performed using AMP and a panel of three AMP analogs that were chosen based on previous literature reports. The bulk of the AMP analogs tested caused a compound-dependent increase in AMPK activity that reached a maximal value before causing a decrease in activity (Fig. **5**). This decrease in activity is due to competition of the AMP analog for the ATP binding site, such as has been observed by others [17, 35]. When the activation assays were repeated in the FRET-based assay using higher concentrations of ATP, the point at which activity began to decrease was shifted to higher concentrations of activator, consistent with competition of the AMP analogs for ATP at the ATP binding site (data not shown).

ATMP, a thiophosphate analog of AMP, was the most potent activator tested in all assay formats, with a half-maximal stimulatory concentration of less than 1 µM (Fig. **5** and Table **1**). It also began to inhibit AMPK at a concentration lower than that observed for the other AMP analogs, inhibiting kinase activity at concentrations above 10 – 100 µM in all formats. When comparing between the two AMPK isoforms, the α_2_β_1_γ_1_ isoform showed half-maximal stimulation at activator concentrations 3 to 6-fold lower than seen for the α_1_β_1_γ_1_ isoform in all assay formats, and comparing between the assay formats for each AMPK isoform, the TR-FRET or CHEF-based assay showed the lowest EC_50_ values, approximately 3- fold lower than the radiometric assay. In addition to being more sensitive to stimulation by ATMP, the α_2_β_1_γ_1_ isoform also showed an approximately 2-fold greater degree of stimulation than the α_1_β_1_γ_1_ isoform. In the radiometric assay, ATMP stimulated the α_1_β_1_γ_1_ isoform 1.7-fold and the α_2_β_1_γ_1_ isoform 2.9-fold, comparable to that seen in the CHEF-based format (1.8- and 2.5-fold for the α_1_β_1_γ_1_ and α_2_β_1_γ_1_ forms, respectively). A greater degree of stimulation for both AMPK isoforms was observed in the FRET-based assay, where ATMP stimulated the α_1_β_1_γ_1_ isoform 3.3-fold and the α_2_β_1_γ_1_ isoform 6.3-fold. Overall, these results compare with those previously reported, in which ATMP stimulated partially purified human AMPK 2.5-fold, and partially purified rat AMPK 3-fold, with a half-maximal stimulatory concentration of 0.65 µM observed for rat AMPK, which then inhibited activity above 25 µM [35].

The physiological activator AMP was the next most potent activator of each AMPK isoform, with half-maximal stimulation occurring at low- to sub-µM concentrations. As with ATMP, there was a slight preference in sensitivity (EC_50_) towards activation of the α_2_β_1_γ_1_ isoform, ranging from 1.1-fold in the radiometric format to 4-fold in the TR-FRET format. Additionally and as with ATMP, the extent to which activity could be increased was greatest for the α_2_β_1_γ_1_ isoform, ranging from 2.5-fold in the radiometric assay to 5-fold in the FRET-based assay. These results are consistent with previous studies comparing activation between the α_1_β_1_γ_1_ and α_2_β_1_γ_1_ complexes in which individual isoforms of rat liver AMPK were immunoprecipitated either from rat liver [36] or from mammalian cells expressing recombinant trimeric rat AMPK [37]. In these previous studies the α_1_β_1_γ_1_ isoform was maximally activated between 1.7- and 3-fold by AMP, whereas α_2_β_1_γ_1_ isoform was maximally activated between 3- and 5.5-fold. However, in these previous studies, the half- maximal concentration of AMP required for activation was 2- to 3- fold lower for the α_1_β_1_γ_1_ isoform when compared to the α_2_β_1_γ_1_ isoform, which is in contrast to our results for the human isoforms in which both isoforms showed nearly identical sensitivity (EC_50_) to AMP in the radiometric format, and 4-fold greater sensitivity towards the α_2_β_1_γ_1_ isoform in the TR-FRET format. Our AMP activation results for the α_1_β_1_γ_1_ isoform are in good agreement with published results using purified recombinant rat α_1_β_1_γ_1_ AMPK, which showed a maximal 3.3-fold activation of AMPK activity, and a half-maximal stimulation at approximately 1.5 µM [30].

The general trends for AMPK activation by ZMP mirrored those seen for ATMP and AMP, albeit with a lower potency than seen for those compounds. Potency was reduced over an order of magnitude relative to AMP against either isoform and in all assay formats, but the maximal stimulation of activity was comparable to that seen with ATMP and AMP, also with slightly higher stimulation and lower EC_50_ values seen for the α_2_β_1_γ_1_ isoform. For either isoform, the maximum increase in activity was seen in the FRET-based assay format relative to the other formats, with the FRET-based assay showing a maximum impact of ZMP on activity of about twice that seen in the radiometric format for either AMPK isoform. Although we are aware of no studies that compare ZMP activation between different AMPK isoforms, both the magnitude of the increase in activity as well as the half maximal stimulatory concentration observed are consistent with data for rat-liver AMPK, with 2-3 fold simulation and half-maximal stimulation occurring between 81 and 164 µM being reported [17, 35].

Finally, the phosphoramidate analog of AMP, APR, was tested as an AMPK activator. The potency of activation was lower than that seen for ZMP in all assay formats except for the CHEF-based format, in which ZMP showed a slightly lower EC_50_ value for activation. With APR there was a more pronounced overall decrease in kinase activity at higher concentrations than was seen for the other activators. Again, both potency and extent of activation was greater for the α_2_β_1_γ_1_ isoform, with the α_1_β_1_γ_1_ isoform showing negligible activation in any formats before inhibiting kinase activity to below basal levels. The maximal extent of activation for the α_2_β_1_γ_1_ isoform (approximately 2 to 3 fold) is comparable to that reported for partially purified human or rat AMPK (2.25-fold and 1.5-fold, respectively). Although it is difficult to estimate the half-maximal concentration of APR required for stimulation from our data, it appears to be greater than 50 µM, which is in contrast to the 1.9 µM value reported for rat AMPK [35].

### Comparison of Results Between Assay Formats

For the radiometric, FRET, and CHEF-based assay formats, each assay was performed at an ATP concentration at or near the apparent ATP K_m_ value as determined in that particular format. Because the response of the TR-FRET format is not strictly linear with respect to product formation (rather, it is dependent on the binding of the phosphopeptide product to the antibody), it is difficult to determine a rigorous K_m_ value for ATP in this format, and the concentration of ATP used in the assay was chosen to match that of the FRET-based assay.

As a general trend, activator EC_50_ values were lowest in the CHEF- and TR-FRET formats. The radiometric and CHEF-based assays showed maximal fold-activation values that were comparable to each other, and approximately 2-fold lower than in the FRET-based assay (in each case the assays were configured such that at maximal activation less than 30% of the substrate would be converted to product, insuring that substrate depletion would have minimal impact on the rate of the reaction). Although the fold-activation was not calculated for the TR-FRET assay due to the non-linear relationship between the TR-FRET value and the extent of substrate phosphorylation, the change in the TR-FRET emission ratio was approximately 2-fold.

The activation data we obtained are consistent with the reported mechanisms for AMP activation of AMPK, in which AMP causes both an increase in the V_max_ of the reaction, as well as a decrease in K_m_ for the peptide substrate [31, 34, 35]. Additionally, the AMP-dependent changes in both V_max_ and K_m_ have been shown to differ substantially when different peptide substrates are used. For example, in radiometric studies using 4 similar AMPK substrates it was observed that the AMP-dependent effect on V_max_ ranged from 2-fold to more than 8-fold, depending on the particular peptide substrate being used in the assay [34]. Moreover, when peptide substrate K_m_ values were determined in the presence or absence of AMP, a 3-4 fold decrease in peptide K_m_ was observed in the presence of AMP when comparing peptide substrates derived from either ACC or HMG CoA reductase [35]. In our radiometric assay the peptide substrate was used at 200 µM, which is above its reported K_m_ value, and the CHEF format used peptide substrate at 8 µM. In contrast, the substrates used in the FRET- and TR-FRET-based assays were used at much lower concentrations, presumably far below the peptide K_m_ value. From the Michaelis-Menten equation:

$$v=\frac{V_{\max}\left[S\right]}{K_{m}+\left[S\right]}$$

it is clear that when [S] > K_m_ (as is the case in the radiometric assay), [S] dominates the denominator, and a decrease in K_m_ for the substrate will have little effect on the rate of the reaction. In contrast, when [S] < K_m_, a decrease in K_m_ will have a larger effect on the denominator, and a greater effect on the rate of the reaction. Thus, in assays to identify AMPK activators in which part of the mechanism of activation is a decrease in substrate K_m_, it may be advantageous to format the assay using sub-K_m_ concentrations of peptide substrate.

The assay formats used in these studies each offer specific advantages and disadvantages relative to one another, and choice of an assay format should depend on the specific goals of the studies being performed. Although radiometric assays are often criticized due to cost associated with waste disposal and regulatory compliance, they typically offer the widest range of substrate flexibility, both in terms of sequence identity and in the concentration of substrate that can be used in the assay. For example, for rigorous studies of enzyme mechanism it is often desired to use one of the substrates at a concentration above its K_m_ value. In a radiometric assay this is limited only by substrate availability (cost) and solubility. In a fluorescence-based assay high concentrations of substrate can be impractical due to inner filter effects (often called “color quenching”) caused by the substrate itself, which results in a decrease in assay signal. In a TR-FRET format, additional complications are encountered at high concentration of substrate due to diffusion-enhanced FRET between donor and acceptor fluorophores. In this case, the high concentration of acceptor fluorophore leads to a greater chance of the acceptor diffusing to within a distance from the donor required for efficient FRET during the time that the FRET signal is measured (typically several hundred microseconds). The result is an increase in background signal, and a decrease in assay window. Although fluorescence-based studies may be performed using high concentration of substrate, they often require high dilutions of the reaction prior to measurement which complicates experimental setup and introduces an additional entry point for errors.

An important characteristic of the FRET- and CHEF-based assays is that the assay output is linear with respect to product formation. This is in contrast to the TR-FRET based format, in which assay response is dependant on binding of phosphorylated product to a phosphospecific antibody. This can make the FRET- and CHEF-based formats preferred for mechanistic studies (with the caveats noted above). Additionally, because the CHEF-based format can be monitored in real time, additional information can be readily obtained for so-called “slow binding” inhibitors. Although not shown here, we observed no lag time (within seconds) for AMP activation of AMPK using the CHEF-based assay.

The TR-FRET format is ideally suited to high-throughput screening of large compound libraries. Because the format is “time-resolved”, optical interference from fluorescent or precipitated compounds (which scatter light) is largely decayed prior to the measurement of the assay signal (in this study, 100 µS after excitation). Like the FRET-based format, the ratiometric nature of the measurement also corrects to a large extent for compounds that absorb excitation light, or for minor variations in well-to-well assay volume.

It is interesting to note that all of the AMPK activators tested showed inhibition of activity at high concentration due to competition for ATP at the enzyme active site. As a consequence, when screening large libraries for AMPK modulators, potent activators could be missed when screening at a single compound concentration. Ideally, screens for AMPK activators should be performed at multiple concentrations of compound (i.e. quantitative HTS or “qHTS” format) so that such activity is not overlooked [38].

## CONCLUSIONS

Although detailed studies of AMPK activity against optimized synthetic peptides have been reported [39], AMPK acts on a variety of substrates, both *in vitro* and *in vivo* (in the liver alone, at least 9 substrates for AMPK have been identified) [40]. When assaying highly purified recombinant AMPK (in contrast to kinase partially purified from tissues or cells), specificity of the peptide substrate for a single kinase is less of a concern than when assaying kinase isolated from cell or tissue samples, and substrate choice can be made based upon the performance of a particular substrate in a given assay format. In this report, we have demonstrated the ability of a variety of artificial peptide substrates to efficiently serve as substrates for AMPK, and demonstrated that the α_2_β_1_γ_1_ isoform is more sensitive towards AMP and AMP-mimetics than is the α_1_β_1_γ_1_ isoform, and that it can be activated to a greater extent. Additionally, in the course of this work we have developed and described in detail the preparation of recombinant trimeric AMPK from insect cells. Taken together, this work should prove useful in the identification of small molecule probes that will facilitate not only a better understanding of AMPK, but in the development of small molecule therapeutics that target this kinase.

## Figures and Tables

**Table 1:** Summary Comparison of AMPK Isoform Properties. n.d. = Not Determined, n.a. = Not Applicable

		ATP Km (µM)		Inhibitor				
AMPK Isoform	Assay Format	- AMP	+ AMP (100 µM)	IC_50_(nM)	Activator EC_50_(µM) / Max. fold-activation			
				*Cpd C*	*AMP*	*ZMP*	*APR*	*ATMP*
α_1_β_1_γ_1_	Radiometric	34	74	230	1.6 / 1.7-fold	165 / 1.5-fold	n.d. / n.a.	0.7 / 1.7-fold
α_1_β_1_γ_1_	TR-FRET	n.d.	n.d.	700	1.4 / n.a.	74 / n.a.	722 / n.a.	0.25 / n.a.
α_1_β_1_γ_1_	FRET	35	83	320	2.5 / 2.8-fold	112 / 3.3-fold	n.d. / n.a.	0.35 / 3.3-fold
α_1_β_1_γ_1_	CHEF	31	25	343	0.6 / 1.8-fold	36 / 1.8-fold	n.d. / n.a.	0.25 / 1.8-fold
α_2_β_1_γ_1_	Radiometric	65	73	88	1.4 / 2.5-fold	54 / 2.5-fold	194 / 1.9-fold	0.13 / 2.9-fold
α_2_β_1_γ_1_	TR-FRET	n.d.	n.d.	500	0.35 / n.a.	23 / n.a.	43 / n.a.	0.04 / n.a.
α_2_β_1_γ_1_	FRET	165	154	140	1.4 / 5-fold	40 / 6.3-fold	n.d. / n.a.	0.10 / 6.3-fold
α_2_β_1_γ_1_	CHEF	82	55	184	0.23 / 2.5-fold	40 / 2.2-fold	n.d. / n.a.	0.07 / 2.5-fold
